# Alpha globin gene alterations modifying the phenotype of homozygous beta thalassaemia

**DOI:** 10.1002/jha2.923

**Published:** 2024-05-27

**Authors:** Jyoti Shaw, Abhilipsa Patra, Anjumana Khatun, Rudra Ray, Amit Ghosh, Sonali Mahapatra, Ashutosh Panigrahi, Maitreyee Bhattacharyya

**Affiliations:** ^1^ Institute of Hematology and Transfusion Medicine (IHTM) MCH Kolkata India; ^2^ Department of Physiology All India Institute of Medical Sciences (AIIMS) Bhubaneswar India; ^3^ Medical Oncology and Hematology All India Institute of Medical Sciences (AIIMS) Bhubaneswar India

**Keywords:** alpha deletion, alpha‐triplication, beta‐thalassemia, co‐inheritance, homozygous

## Abstract

The phenotype of β‐thalassemia varies widely. The primary determinant is the type of beta‐globin gene mutation; however, there are secondary and tertiary modifiers also as associated alpha mutations, polymorphisms, as well as coinheritance of mutations affecting other related systems. Co‐inheritance of alpha thalassemia mutations is known to ameliorate the severity of HbE‐β thalassemia. However, the role of alpha globin gene alterations (deletions and triplication) is not well illustrated in homozygous β‐thalassemia. Here we evaluated the role of alpha globin gene alterations in 122 β‐thalassemia patients having IVS1‐5 (G > C) homozygous mutation. β‐thalassemia mutations were detected by ARMS PCR and alpha mutations by GAP‐PCR. Gene expression by qRT‐PCR. Out of 122 cases, 15 patients had alpha 3.7 triplications (ααα^3.7anti^), 24 had alpha 3.7 kb deletion (−α^3.7^) mutation and three patients had 4.2 kb deletion (−α^4.2^). Patients were divided into two groups, requiring less than 8 units (NTDT) and more than 8 units (TDT) of blood transfusion per year (≥8U BT/year). The percentage of alpha deletion was significantly (*p* = 0.0042) high in NTDT (42.1%) as compared with TDT (13.2%). Conversely, the proportion of alpha triplication is high in the TDT as compared with NTDT. Even mean serum ferritin level was found to be significantly high in patients having alpha triplication as compared with those having alpha deletions (*p* = 0.0184) and normal alpha gene (*p* = 0.0003). α/β globin ratio was highest in TDT patients with alpha triplication and lowest in NTDT patients with alpha‐del. The results show that concurrent inheritance of alpha gene alterations influences the phenotypic severity of homozygous β‐thalassemia.

## 1 INTRODUCTION

Beta‐thalassemia (β‐thal) is the second most common hemoglobinopathy in the world after alpha thalassemia and is the major health problem implying a huge burden upon society and country [1]. In India, the prevalence of β‐thal carrier ranges from 4% to 17% among different states and every year 10,000–12,000 children are born with the disease [2]. Pathophysiology of the disease lies in the imbalance between alpha and beta globin chains. Severity of the disease increases when the ratio of alpha/beta globin chain is high.

Primary factors governing this alpha‐to‐beta chain ratio (α:β) are the type of beta gene mutation like beta zero (β^0^) or beta plus (β^+^) as well as concurrent inheritance of alpha thalassemia (α‐thal) mutations which reduces the expression of alpha chains. Large deletions like –SEA; ‐THAI; ‐MED; −3.7 kb; −4.2 kb; −5.3 kb, ‐SA; in alpha globin gene are more common in α‐thal group [3]. A systematic review by Pallavi et al. [4] on molecular heterogeneity of HbH in the Indian population showed high prevalence of −3.7 kb, −4.2 kb, and –SA deletion in the alpha‐globin gene as compared with other deletion mutations, out of which 3.7 and 4.2 kb deletions most commonly reported from Indian population in different regions of the country and 3.7 kb deletion is the commonest of all [5]. An earlier report by Nadkarni et al 1996 on 100 normal and 230 β‐thal carriers showed a high frequency of −3.7 kb [6].

Alpha globin gene cluster comprises of two active alpha genes (alpha 2 and alpha 1) and both are expressed throughout the life of an individual. Alpha 2 and alpha 1 genes are further subdivided into homolog boxes like (X, Y, and Z). At the time of genetic recombination during meiosis, unequal crossover and misalignment between these homolog boxes (Z1 and Z2) result in alpha 3.7 kb deletion in one allele and alpha 3.7 triplication in the other allele and similarly crossover between homolog boxes X1 and X2 result in alpha 4.2 deletion and 4.2 triplication. [7]

Earlier studies on HbE‐β thalassemia from our center also showed a greater prevalence of 3.7 kb deletion. [8, 9] Co‐inheritance of alpha deletions in HbE‐β thalassemia were found to ameliorate the clinical severity of patients. There are studies which showed the co‐inheritance of alpha thalassemia determinant in β thalassemia but in a small series of patients and beta gene mutation was heterogenous [10] The type of beta mutation also govern the α:β globin chain imbalance. Hence to overcome the variation in severity due to beta mutation, in the present study, a homogenous population of β thalassemia was selected. Here, we comprehensively studied a homogenous population of β thalassemia harboring IVS1‐5(G > C) in a homozygous state and investigated the role of alpha globin gene alterations in modifying the phenotypes in them.

## 2 MATERIALS AND METHODS

### 2.1 Subjects

The study population comprised the β‐thal patients who attended the Thalassemia Outpatient Department (OPD) of our institute. Based on the HPLC report homozygous β‐thal cases were selected. A detailed history and thorough clinical examination were done for each patient. Informed consent was taken from each patient prior to including them in the study. A blood sample was collected in EDTA and plain vials for further molecular and biochemical analysis. Patients who received transfusion less than 15 days before sample collection were also excluded from the study.

### 2.2 Hematological parameters

A part of the peripheral blood was subjected to complete blood count in Sysmex XP‐100 for analysis of complete blood count.

### 2.3 Serum ferritin

Serum ferritin level was assayed by ELISA method using an Accubind ferritin kit in a 96‐well microwell plate. The absorbance was read at 450 nm in a microplate reader.

### 2.4 ARMS‐PCR

ARMS PCR was performed for the detection of IVS1‐5(G > C) point mutation in the DNA samples of the patients following the primers and protocol described in Roshan Colah et al. [11].

### 2.5 GAP‐PCR

3.7 kb deletion or triplication and 4.2 kb deletion or triplication are the most alpha globin gene alterations reported from the Indian population. These two mutations in the alpha globin gene were detected by the GAP–PCR method. Wang et al. [12] protocol was followed for alpha triplication (ααα^3.7 anti^ and ααα^4.2anti^) and Liu et al. [13] method was followed for the detection of alpha globin gene deletions (−α^3.7^/−α^4.2^). Alpha globin double deletion in cis form was investigated in patients’ DNA which gave negative results in the GAP‐PCR reaction. Double deletion in cis form in alpha gene is determined by multiplex PCR followed by Tan et al. [14].

### 2.6 Globin gene expression

RNA was isolated from fresh blood samples using a Qiagen RNA isolation kit following manufacturer protocol and cDNA was prepared using RT2 First strand kit from Qiagen. 0.5 µg of RNA was used for cDNA synthesis of each sample. cDNA was stored in a deep fridge before usage.

### 2.7 Realtime PCR

Gene expression study was done for the alpha 2 globin gene (HBA2) and beta‐globin gene (HBB). Primers were designed using the IDT qPCR primer tool spanning the junction of two exons. Realtime PCR was performed using SYBR Green Master Mix procured from Qiagen and β‐actin gene kept as internal control gene. Comparative CT method (ΔΔCT) was used for data analysis.

### 2.8 Statistical analysis

Data were analyzed in MS Excel and GraphPad statistical programs. Unpaired *t*‐test was performed to compare the mean between groups and *p*‐value < 0.05 at a 95% Confidence interval was considered significant. Genefold expression of the HBA2 gene with respect to the HBB gene was compared using ΔΔCT and data was shown as 2^(−ΔΔCT)^ in the bar graph.

## 3 RESULTS

### 3.1 Demography of the subjects

Six hundred seventy‐six thalassemia patients from OPD were screened and out of these 310 patients were homozygous beta thalassemia and rest 366 were others like HbE‐Beta, HbS‐Beta, etc. Among the 310 beta thalassemia cases, IVS1‐5(G > C) homozygosity was found in 122 cases. The demography and baseline laboratory parameters of the subjects were summarized in the table below (Table 1). All the patients were on regular chelation with deferasirox as per the departmental protocol. The age of the patients varied from 1.2 to 34 years with mode of 11. Only 14 patients (11.4%) aged above 18 years and one patient was 34 years old. The average age of diagnosis was 3.9 but 61% of cases (74) were diagnosed below 2 years of age.

**TABLE 1:** Demography and laboratory parameters of the study population.

Parameter	Average	Range
Age (years)	11.3	1.2–34
Sex	66 males	56 females
Age of diagnosis (years)	3.9	1–24
Age of first transfusion (months)	14.43	1–132
Frequency of transfusion (units/year)	12.12	2–48
Spleen size (cm)^a^	3.46	0–15
Haemoglobin (g/dL)	6.93	3–12
Serum ferritin (ng/mL)	1027	86.9–1650
HbF (%)	20.59	0–106.8
Pubertal development	Delayed	

^a^ Spleen and liver were physically measured by the clinician below the costal margin.

### 3.2 Distribution of alpha globin gene mutation in the IVSI‐5 (CC) β‐thalassemia

**FIGURE 1:**
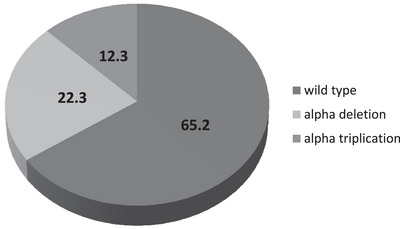
Distribution of alpha globin gene mutation in the β‐thal homo patients. The pie chart shows the distribution of alpha globin gene deletion/triplication among the homozygous β‐thal patients.

The overall distribution of alpha globin gene mutation is shown in Figure 1. 12.3% (*n* = 15) of the population possessed 3.7 kb alpha triplication (ααα^3.7anti^). None of the cases had 4.2 kb triplication (ααα^4.2anti^). 22.3% (*n* = 27) cases possessed alpha deletion for either 3.7 or 4.2 kb. Twenty‐three patients had 3.7 kb single deletion (αα/α^−3.7^), and two patients had 4.2 kb single deletion (αα/α^−4.2^). One patient had 3.7 kb double deletion (α^−3.7^/α^−3.7^) and another patient was heterozygous for alpha deletion having both 3.7 and 4.2 kb deletion (α^−3.7^/α^−4.2^). One patient co‐inherited both 3.7 kb deletion and 3.7 kb triplication (ααα^3.7anti^/α^−3.7^). None of the patients had a double deletion of the alpha globin gene in cis form as checked by multiplex PCR described by Tan et al. [14].

### 3.3 Influence of alpha globin gene alterations on the Hb Level of the β‐thal patients

**FIGURE 2:**
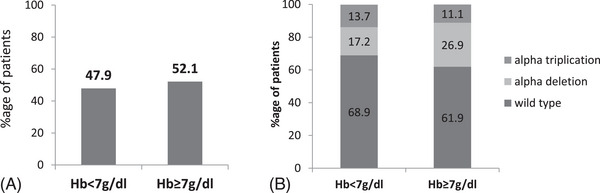
Comparison based on the baseline Hb level. (A) Bar‐graph shows the percentage of patients having baseline Hb ≥7 g/dL or Hb < 7 g/dL. (B) A composite bar diagram of the percentage distribution of alpha globin gene mutation among the two levels of baseline Hb.

Subjects were divided into two categories of baseline hemoglobin levels. One category comprised cases that had Hb < 7 g/dL and other had Hb≥7 g/dL. 47.5% (58) of the cases had Hb < 7 g/dL whereas 52.4% (64) had Hb≥7 g/dL. Almost half of the subjects maintained Hb below 7 g/dL. The minimum baseline Hb found was 3 g/dL and the maximum at 12 g/dL (Figure 2A). Out of 122 patients, only two patients had average baseline Hb 3 and 3.9 but one of them received 7 units of BT/year and other received 12 units of BT/year, respectively. These two patients failed to kept records of their pretransfusion and only one or two data were available for 1 year. If all the values of Hb were present, then the average Hb for these patients must be greater.

Figure 2B shows the distribution of alpha mutation amongst the Hb < 7 g/dL and Hb≥7 g/dl groups. A higher percentage (13.7%) of patients with alpha triplication had hemoglobin less than 7 g/dL. In contrast, higher percentage (26.9%) of patients with alpha deletion maintained hemoglobin levels greater than 7 g/dL.

### 3.4 Influence of alpha globin alterations on the transfusion frequency of the β‐ thal patients

**FIGURE 3:**
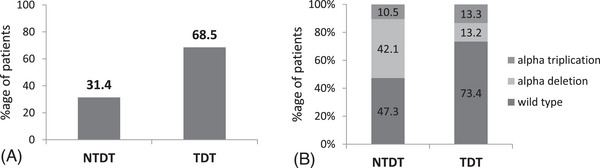
Comparison between NTDT (BT < 8/year) and TDT (≥8/year) β‐thal patients. (A) Bar‐graph shows the percentage of NTDT and TDT patients. (B) The composite bar diagram of the percentage distribution of alpha globin gene mutation among the two groups.

The subjects were re‐assessed into two arms based on their blood transfusion (BT) requirement (Figure 3A). Those requiring less than BT 8U/year were NTDT and those requiring more than BT 8U/year were TDT [8]. It was found that 31.4% (38) of cases required less BT whereas 68.5% (84) needed more BT per year. Mean BT/year for the whole population was 12.12 BT units with a maximum of 48 and minimum of 2U BT/year.

Figure 3B shows the distribution of alpha mutation among the NTDT and TDT. Transfusion frequency analysis revealed that a significantly (*p* = 0.0042) higher number of patients (42.1%) with alpha deletions required lesser BT per year as compared with those patients (13.2%) who needed more BT/year with the same genotype. However, co‐inheritance of alpha triplication increased the dependency on transfusion as 13.3% with alpha trip needed more than 8U BT per year as compared with 10.5% of alpha trip who required less transfusion. Thus, alpha deletion reduced the clinical severity whereas alpha triplication increased the severity of β‐thal patients.

### 3.5 Influence of alpha globin alteration on globin gene imbalance in β‐thal patients

**FIGURE 4:**
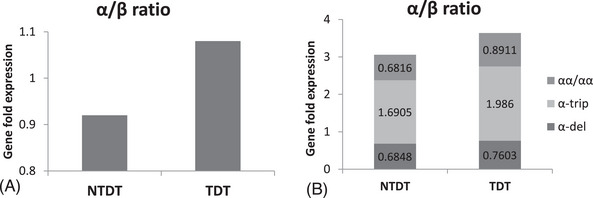
Relative quantification of alpha globin gene. (A) Bar‐graph shows the genefold expression (2^−∆∆CT^) between NTDT and TDT. (B) The composite bar diagram of genefold expression among the different genotypes of the alpha globin gene.

qPCR data of alpha gene 2 (HBA2) and HBB with respect to β‐actin gene expression was at par with the data of Cristina Zuccato et al. [15], who studied globin gene expression in β‐thal patients. HBA2 gene expression was compared with the HBB gene in these IVS1‐5(CC) β‐thal patients and data was shown as 2^(−ΔΔCT)^. Figure 4A shows the α/β ratio in NTDT and TDT groups. In NTDT, α/β ratio is less as compared with TDT patients. On further analyzing gene expression in three subgroups of alpha genotype, it was found that α/β ratio was highest in alpha trip in TDT patients and lowest in the alpha‐deletion group and with wild type alpha gene. Figure 4 shows that alpha triplication and deletion had a direct impact on the gene expression of the alpha globin gene which further modifies the phenotypes in these patients.

### 3.6 Comparison of clinical features of β‐thal between α‐trip and other two genotypes of alpha globin gene (α‐del, αα/αα)

Table 2 compares some important clinical features of β‐thalassemia which defines the severity of the disease among the three genotypes of the alpha globin gene. Disease presentation was found to be early in the α‐trip as compared with α‐del group. The mean age of 1st BT was lowest (*p* = 0.038) in alpha trip as compared with alpha del and wild type. Serum ferritin and spleen size (physically measured by palpation below the costal margin) were greater in alpha trip as compared with the other two groups. Ferritin level was found to be significantly higher in alpha trip group as compared with alpha deletion (*p* = 0.0184) and wild type (*p* = 0.0003) groups. β‐thal patients with ααα^3.7anti^ were more severe as seven out of 15 had splenectomy and seven had growth retardation like delayed puberty. In the wild‐type alpha group, 10 out of 80 were splenectomised. The alpha/beta globin ratio was highest in α‐trip group and lowest in the α‐del group.

**TABLE 2:** Comparison of clinical features among three genotypes of alpha globin gene.

Parameters	α trip (*n* = 15)	α del (*n* = 27)	αα/αα (*n* = 80)
Mean age_diagnosis (year)	4.85 ± 1.2	5.13 ± 0.77	3.26 ± 0.47
Mean age_1st BT (months)	8.67 ± 2.06	20.2 ± 4.37	11.92 ± 1.6
Baseline Hb (g/dL)	6.84 ± 0.21	7.1 ± 0.22	6.91 ± 0.18
Mean BT/year (units)	10.72 ± 1.34	9.11 ± 1.14	13.29 ± 0.91
Mean serum ferritin (ng/mL)	1372 ± 151.8	934.4 ± 102.6^a^	1044.6 ± 25.09^a^
Mean HbF (%)	16 ± 8.05	20.37 ± 5.55	20.67 ± 3.61
Spleen size (cm)^b^	5	3.8	3.27
Liver size (cm)^b^	2.83	3.25	2.98
Splenectomised	7	0	10
Growth retardation	6	7	23
Alpha/Beta globin ratio	1.838	0.722	0.786

^a^ The difference is statistically significant as compared with the alpha trip group.

^b^ Spleen and liver were physically measured by the clinician below the costal margin.

## 4 DISCUSSION

IVS1‐5 (G > C) is the second most prevalent β globin gene mutation after CD 26 (G > A) (HbE) in India varying from 15% to 88% in different parts of the country [16, 17]. β‐thal patients harboring (IVS1‐5 (G > C)) in homozygous state are considered to behave as thalassaemia major. However, in 122 β‐thal (IVS1‐5(CC)) patients studied, wide variations in phenotypes and clinical presentation were noticed. Table 1 summarizes the clinical features of the patients investigated. Average age of subjects was 11.3 years inspite of wide range in age (1.2–34) as the mode age is 11 years. Thus, height and weight among the patients could not be compared. HbF values represent data at age of diagnosis. 61% (74) of patients were diagnosed below 2 years of age and thus HbF values were high and only 8% of cases were diagnosed above 10 years. There is still a huge difference between minimum and maximum values in case of Hb level, age of diagnosis, age of presentation, transfusion frequency, and ferritin level which suggest a wide variation in phenotype. Hence to understand this wide difference in phenotypic severity, we further investigated alpha globin gene alterations. Co‐inheritance of alpha thalassemia is known to ameliorate clinical presentation in HbE‐β thal heterozygotes [8, 17, 18].

In 122 (IVS1‐5(CC) homo β‐thal studied, 22.3% possessed which is slightly lower as compared with HbE‐β thal patients from our center which were shown to carry α‐deletion at the range of 24.5–25.1%. [8, 18] However, lower prevalence of α‐deletion (12.9%) among HbE‐β‐thal was reported from Delhi. [17] Nadkarni et al. [10] from Maharashtra reported 21.8% prevalence of α‐deletion in 64 β‐thal patients which was almost similar to our data.

A recent study from Greece described the mutation basis in 217 patients with hemoglobinopathy and its impact on disease severity. It also revealed higher frequency of α‐deletion among TM as compared with α‐triplication. They concluded that co‐inheritance of α−3.7 among 15 TM increased haemoglobin level reduced severity whereas double heterozygotes with α‐triplication and TI presented with a more severe phenotype. However, no frequency or rate was mentioned in the study [19].

Lower prevalence of α‐triplication is also reported from other Asian countries. Iranian population reported α‐triplication in the range of 1.2–1.67% among β‐thal carriers [20, 21]. The Sri Lankan cohort composed of 620 severe to moderate β‐thal patients had a concurrent inheritance of 2% α‐triplication [22]. Variable frequency of α‐triplication is found in the Indian population from different regions. 1.6% α‐triplication is found in 311 samples from Gujarat and 1% in 510 samples from Maharashtra [5]. However, the prevalence of α‐triplication is much higher among β‐thal compared with the general population. In the present study, we found 12.3% of α‐triplication (ααα^3.7anti^) in 122 (IVS1‐5(CC) homo β‐thal. In another study by Mehta et al in 178 β‐thal carriers behaving as thalassemia intermedia, α‐triplication was found in 20.2% of cases [23]. On the contrary, the frequency of α‐triplication among HbE‐β‐thal patients from our center was found to be very low at 0.9% [17]. This needs further evaluation which is beyond the scope of the present study.

Alpha triplication is mostly studied in β thal carriers having one β globin gene affected but behaving as intermedia [20, 21, 23]. Mehta et al. [23] found α‐triplication in three β thal patients having β^0^/β^+^ or β^++^and 26 β thal carriers but having severe clinical presentation out of 181 patients studied, whereas Farashi et al. [19] found α‐triplication in 14 β thal carriers and 9 β‐thal major out of 1700 patients studied. None of them commented on the influence of α‐triplication on disease severity. Another retrospective study from Greece on 24 β‐thal heterozygotes, showed that co‐inheritance of α‐triplication results in more severe symptoms due to menstrual cycle and pregnancy [24].

Numerical aberration in the α‐globin gene in β‐thal (IVS1‐5(CC)) is found to modulate disease severity. Patients with α‐triplication had more severe clinical features as compared with the ones having α‐deletion or normal α‐globin gene. All the parameters like age of diagnosis, age of first transfusion, frequency of BT, baseline Hb, mean HbF, and Ferritin are better in patients with alpha deletion; however, none of these were statistically significant except serum ferritin (*p* = 0.0184).

To understand the influence of α‐globin gene alterations, we divided the patients into two groups based on Hb level, we found 47.5% of patients having Hb below 7 and 54.2% having above 7 g/dL. The proportion of α‐triplication was higher in the group having Hb < 7 (13.7%) as compared with the group having Hb > 7(11.1%); however, it was not statistically significant. On the contrary, α‐deletion was found in comparatively higher proportion in the groups having Hb > 7 g/dL (26.9%). Thus, the presence of alpha triplication increases the α/β globin chain imbalance whereas presence of alpha deletion reduces the α/β globin ratio. Gene expression study (Table 2) shows that α/β globin ratio of the alpha trip group is highest in both NTDT and TDT groups and α/β globin ratio is lowest in alpha deletion group of β‐thal patients. Thus, the lesser the globin gene imbalance, the longevity of RBCs increases and improves Hb level in β‐thal patients.

β‐thal patients are defined as thal major when require regular blood transfusions for their survival. The degree of severity is often defined by the number of transfusions required per year. Hence in the present study, we again divided the subjects into two arms based on transfusion requirement per year—one requires BT < 8 U/year (NTDT) and another requires BT≥8 U/year (TDT). 31.4% are NTDT and 68.5% are TDT. Alpha globin gene investigation showed that a significantly (*p* = 0.0042) higher percentage of patients (42.1%) with α‐deletion are in the NTDT arm as compared with 13.2% in TDT. The reverse is found in the case of α‐triplication, a higher percentage (13.3%) was found in the TDT arm and a lower percentage (10.5%) was in the NTDT arm. Globin gene expression result shows that α/β ratio was highest in the TDT group with α‐triplication (1.986) and lowest in NTDT with α‐deletion (0.684) and NTDT with wild type α gene (0.681) (Figure 4). Hence, quantitative PCR data further validates the hypothesis that the presence of α‐globin gene determinant has direct impact on globin chain imbalance and thus influences disease severity.

In the present study, we comprehensively investigated α‐globin alterations and illustrated its influence on the clinical phenotypes in a homozygous population of β‐thal having (IVS1‐5(CC)). A similar study was reported by Siriworadechkul et al. on β‐thal /HbE disease patients in 2014. [25] Even they showed the co‐inheritance of α‐thalassemia trait ameliorate disease severity but influence of α‐triplication was not investigated in their study. This study clearly shows the presence of α‐triplication makes the clinical phenotype more severe and reverse is true for α‐deletion. However, there are few limitations like the number of the study population in all three categories of α‐globin genotype was not equal and majority of the population (65.5%) possessed normal α‐globin gene numbers but had variable clinical features. This needs further investigation of other secondary and tertiary modifiers of severity in β‐thal major.

## 5 CONCLUSION

Alpha gene deletion or triplication alters the clinical phenotype of β‐thal patients. Detecting alpha globin gene mutation status will help to predict the severity of phenotype.

## AUTHOR CONTRIBUTIONS

Jyoti Shaw performed the qPCR experiments, analyzed the data, and wrote the manuscript. Abhilipsa Patra performed the alpha mutation study. Anjumana performed the beta mutation study and helped in the alpha mutation study. Rudra Ray initiated the study. Amit Ghosh guided A.P. Sonali Mahapatra and Ashutosh Panigrahi recruited subjects and clinically examined the patients in AIIMS Bhubaneswar. Maitreyee Bhattacharyya recruited subjects and clinically examined the patients in IHTM and gave valuable insights and edited the manuscript.

## CONFLICT OF INTEREST STATEMENT

The authors declare no conflict of interest.

## ETHICS APPROVAL STATEMENT

The study was duly approved by the Institute Ethics Committee (IEC), Medical College Kolkata. IEC approval number is MC/KOL/IEC/NON‐SPION/142/09‐2018.

## PATIENT CONSENT STATEMENT

Written informed consent was taken from the patients.

## CLINICAL TRIAL REGISTRATION

The authors have confirmed clinical trial registration is not needed for this submission.

## Data Availability

Data were taken from the thalassemia patients registered in IHTM and attending thalassemia clinics. The data will be available from the corresponding author upon reasonable request.
